# Fracture and deformation of ProTaper Next instruments after clinical use

**DOI:** 10.4317/jced.54910

**Published:** 2018-11-01

**Authors:** Gema Fernández-Pazos, Benjamín Martín-Biedma, Purificación Varela-Patiño, Manuel Ruíz-Piñón, Pablo Castelo-Baz

**Affiliations:** 1 Department of Pathology and Dental Therapeutics II, Universidad de Santiago de Compostela, Galicia, Spain

## Abstract

**Background:**

The aim of this study was to evaluate the fracture and defects observed in ProTaper Next files discarded after a normal clinical use.

**Material and Methods:**

571 ProTaper Next rotary instruments were collected after clinical use from the clinic of endodontics over 12 months. The length of the files was measured using a digital caliper to determine any fracture, later all the files were evaluated under a stereomicroscope to observe defects such as unwinding, curving or fracture. The data obtained were analyzed using a chi-square and z test.

**Results:**

13.83% of the discarded files showed defects, the most frequent defect was fracture (7.53%). The highest rate of fracture was observed in the X1 (17.04) files (19.87%). The presence of deformations without fracture was also more frequent in the 17.04 file (11.8%).

**Conclusions:**

Because of the relatively high incidence of deformation of smaller files, these instruments should be considered as a single use. It is important not to exceed the maximum uses recommended by the manufacturer to reduce the risk of cyclic fatigue, the main cause of fracture of the files (79.07%). It is also important to observe each file after use to discard small defects or fractures.

** Key words:**Clinical use, deformation, fracture, M-Wire, ProTaper Next.

## Introduction

The fracture of instruments within the canal is one of the most frequent complications of endodontic treatment which can adversely affect treatment outcome (1,2). In general, the fractured instruments hide or block access to the apical portion of canal and therefore compromise the effectiveness of cleaning and shaping procedures of the same (3).

The fracture of rotary nickel-titanium instruments (NiTi) can occur for different reasons: the fracture caused by torsion, the fracture caused by cyclic fatigue or a combination of both causations (4,5).

Torsional fracture occurs when the tip or another part of the instrument is locking in the canal while file continues rotating. When the elastic limit of the metal is exceeded, the fracture of the tip thereof is inevitable (6,7). The fracture caused by cyclic fatigue occurs due to metal fatigue. The instrument does not adapt to the canal but it freely rotates in the curvature, generating in the point of maximum bending cycles of tension/compression until the fracture occurs. When the instrument is held in a static position and it continues rotating, the midshaft of the instrument that is outside the curvature undergoes tension, while the other half that is on the inside of the curvature undergoes compression (8,9).

The nickel-titanium instruments have had an important development in endodontics; they have reduced operator fatigue, procedural errors, and the duration of the canal preparation. The mechanical properties of nickel-titanium have enabled files to be more flexible, and better to conform curved canals and to resist fracture (10-12).

ProTaper Next (Dentsply Maillefer, Ballaigues, Switzerland) is a group of rotary files designed with a variable taper and with a rectangular cross section decentered. The file set includes five conformation instruments with variable taper: at the tip, X1 17.04, X2 25.06, X3 30.07, X4 40.06 and X5 50.06. With all instruments are expected passively follow the canal to reach the working length.

ProTaper Next is manufactured with the recent alloy of nickel-titanium, M-Wire, prepared by a special thermal process, which aims to improve flexibility and resistance to cyclic fatigue (13-17). With this alloy is expected to have greater resistance and less wear than similar instruments made of conventional superelastic NiTi wires because of its unique nanocrystalline martensitic microstructure (18,19). So far, there are no published studies that evaluates the deformation and fracture of ProTaper Next instruments after clinical use. The aim of this study was to evaluate the fracture and defects observed on ProTaper Next rotary instruments discarded after a normal clinical use.

## Material and Methods

Five hundred seventy-one files of ProTaper Next rotary system used in the last 12 months in the exclusive clinic of endodontics were collected. The files were discarded after a normal clinical use, without any visible defect or many of them due to defects observed as unwinding, curving or bending or, lastly, because of its fracture. Moreover, the clinicians were strictly informed that they could not use each file for the preparation of more than two molars with wide canals or, if its canals were curved, narrow or calcified they should discard them after shaping of each molar. They were also informed that they should use microscope (Zumax OMS 2350, Suzhou New District, China) and examine all the files before each use. The instruments were used by 6 operators, specialist in Endodontics, trained in the use of the ProTaper Next system with the X-Smart motor (Denstply Maillefer, Ballaigues, Switzerland) with recommended settings by the manufacturer, speed: 300 rpm and torque: 4-5.2 Ncm.

To obtain a suitable glide path of all the canals K-files size 08, 10, 15 and 20 were used. The preparation with ProTaper Next rotary system began with the X1 (17.04) file, and finished with the file that indicated the gauging of each canal. All the instruments were used at the full working length as recommended by the manufacturer. The canals were irrigated with sodium hypochlorite 5.25% and EDTA 17%. All files were inspected and sterilized after each use.

The discarded files were grouped according to the file number (17.04 to 50.06). All the files were 25-mm long. To determine if any fracture existed and the location of the fracture, the length of each discarded instrument was measured from the shaft to the tip by using a digital caliper (Mestra, Sondika Bilbao, Spain). Then, each file was inspected under a stereomicroscope (Leica MZ 12.5; Leica, Heerbrugg, Germany) at 25x, 32x and 40x magnifications for other defects such as unwinding, curving or bending. The fractured files were examined longitudinally and categorized into torsional or flexural failure according to Sattapan *et al.* If there was a defect in the fractured files like unwinding, reverse unwinding or tightening of the spirals, it was torsional, if not flexural (10).

Chi-square analysis was applied to analyse whether the fracture and deformation presence of the files statistically depended on the size of the files. On the other hand it was used a z test to determine the difference between the visible and non-visible defects. The significant level was <0.05.

## Results

A total of 571 files of ProTaper Next rotary system were discarded of the clinical practice in the last 12 months. The number and size from discarded files are shown in  Table 1. The most frequently discarded files were X1 17.04 (161 files), X2 25.06 (159 files), and X3 30.07 (155 files).

**Table 1:**
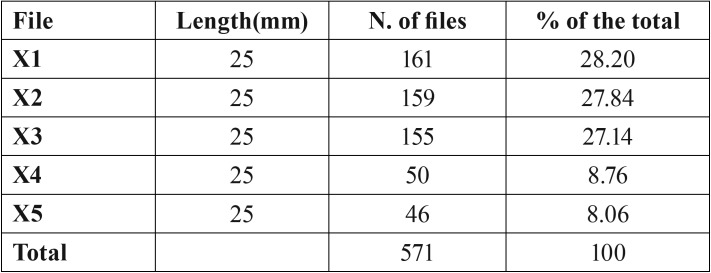
Summary of all discarded files after normal clinical use in endodontic practice in the last 12 months.

The defects detected from discarded files are shown in  Table 2. A percentage of 13.83% of all discarded files shown defects. A chi-square test (X2) was applied to analyze whether the presence of fracture and deformation statistically depended on the size of the files. No statistically significant differences were found (*p*> 0.05), therefore the presence of fracture and deformation does not depend on the size of the files.

**Table 2:**
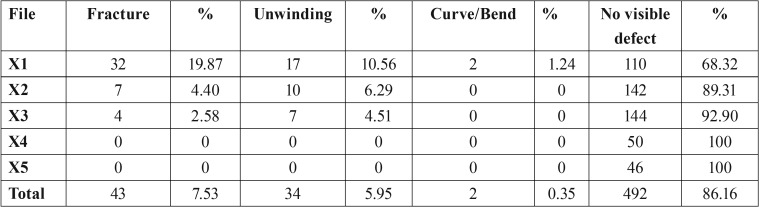
Details of defects observed on discarded ProTaper Next instruments.

The major defect was the fracture of the file (7.53% of all instruments). The highest rate of fracture was observed on the 17.04 files (19.87%), followed by 25.06 (4.40%) and 30.07 (2.58%). The rest of files did not show fractures.

**Figure 1:**
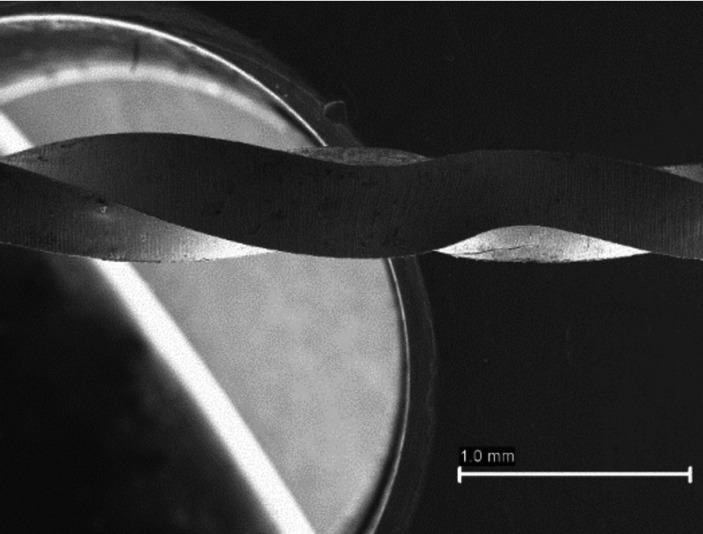
Images captured by stereomicroscope. Unwinding file.

Unwinding (Fig. 1) was also observed mainly in the 17.04 file (17 files, 10.56%), like the presence of curving or bending (2 files, 1.24%). This caused the file X1 presented the highest rate of defects (31.67%) followed by the file X2 (10.69%). It is important to note that the X4 and X5 files did not show any type of defect. On the other hand, a z test was realized to check the difference between visible and non-visible defects, and the defects are significantly different (*p*<0.01).

**Figure 2:**
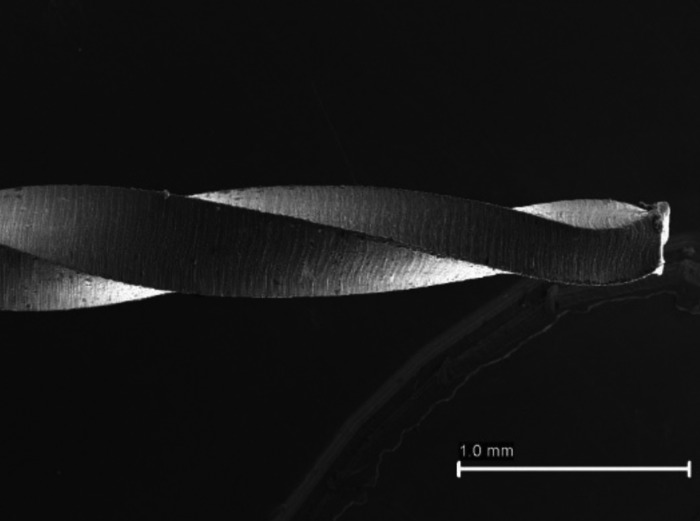
Images captured by stereomicroscope. Fractured file by torsional.

**Figure 3:**
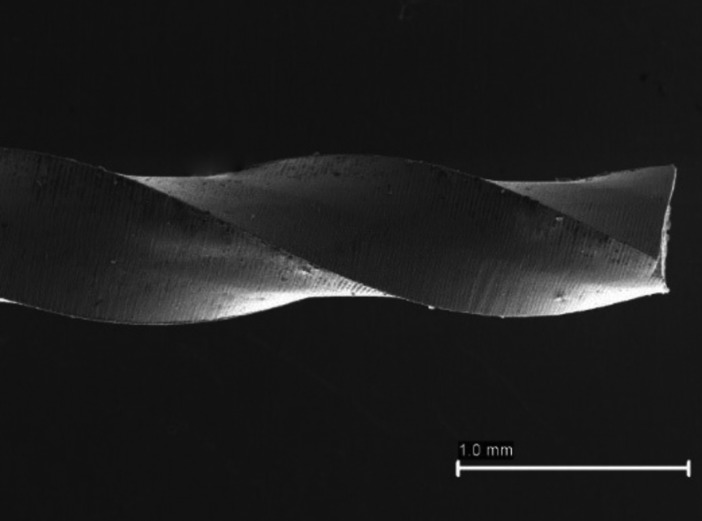
Images captured by stereomicroscope. Fractured file by cyclic fatigue.

Fractured instruments were divided according to the type of fracture, torsional (Fig. 2) or flexural fatigue (Fig. 3), the number and percentage of files of each group are shown in  Table 3. The stereomicroscope evaluation revealed the reason for the separation of 34 (79.07%) files was flexural fatigue and 9 (20.93%) files were torsional. Both types of fracture were observed more often on X1 file.

**Table 3:**
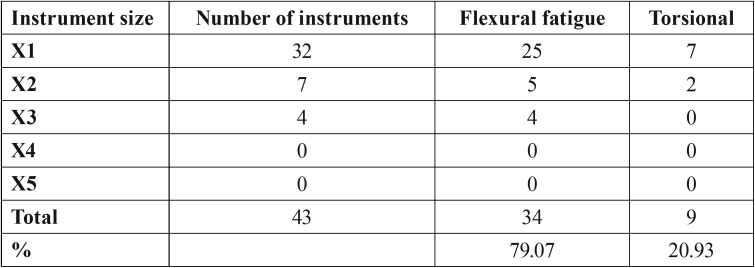
Analysis of fractures.

Based on the initial length of the files was 25 mm, the fragment size ranged from 4.27 to 1.29 mm. The fractured instruments due to cyclic fatigue are generally larger.

## Discussion

The aim of this study was to evaluate the presence of deformation and fracture of rotary instruments ProTaper Next manufactured with the NiTi alloy, M-Wire and discarded after normal clinical use. The first three files (17.04, 25.06, and 30.07) of the rotary system were the most discarded. This was expected because of the complete sequence is not always necessary to use, since the diameter of the tip that must have the last rotary instrument that we used in each canal was indicated by the smallest diameter of the apical constriction. Therefore, these files of ProTaper Next system were used by clinicians more frequently than the others.

In this study, a high percentage of fracture and/or deformation was observed on the smallest instruments. Similar to our results, other authors have been reported that smaller instruments were more likely to show distortion and separation than the larger instruments (20-22). Inan *et al.*, studied the deformation and fracture of Mtwo rotary nickel-titanium instruments and they concluded that the magnitude of stress on the Mtwo instruments with smaller size would be higher (11). In 2009, a study suggested that small size files should be considered as single-use, disposable instruments because of the higher possibility of deformation and fracture (22).

Sattapan *et al.* disclosed two main features of fractured instruments manufactured with conventional nickel-titanium (10). One feature was the demonstration of a visible defect associated with the fracture: unwinding, reverse spires, reverse spires with stretching thereof or a combination of the three defects. The other feature was the fracture without any visible defect to accompany it. The authors considered that these two types of fractures were caused by different mechanisms: torsional fracture and fracture caused for flexural fatigue of the alloy. This is based on their experiment that involving fracture of the instruments under simulated conditions of dynamic torsional and bending. The results showed a consistent pattern for each type of fracture. All instruments subject to torsional fracture showed similar defects, while instruments fractured for bending showed an abrupt fracture without any defect accompany them. The aforementioned criteria, the fracture with or without deformation of instruments, helps classify the broken files into two main groups. In the classification used in our study the cause of the fracture is identified and this could lead to change in clinical use to prevent file fracture.

Using the above criteria, our results showed that more instruments fractured by bending (79.07%) than by torsioning (20.93%). In 2007, a study evaluated ProTaper instruments, made of conventional nickel-titanium alloy discarded after clinical use, and it was observed that the most instruments fractured due to flexural fatigue and not to torsional (23). Although in our study metal fatigue was the predominant cause for fracture, the smaller instruments (17.04, 25.06 and 30.07) showed more torsional fracture than larger diameter instruments. This was an expected result because the smaller diameter files are more likely to lock at the tip and to fracture with less torque.

Al-Hadlaq *et al.* evaluated fatigue by cyclic bending of rotary files GT Series X manufactured with the M-Wire alloy and compared them with GT and Profile made of conventional nickel-titanium alloy (24). They observed that nickel-titanium rotary files manufactured with the M-Wire alloy have better fatigue cyclic resistance than files with similar design and size manufactured with nickel-titanium conventional alloy. Therefore, with this alloy a more resistant files to a fracture are achieved.

We found that most of the 17.04, 25.06 and 30.07 files fractured in the apical third, and the length of the fractured fragment was 4.27 mm or less. Many of these fragments were so small that the clinician might not be aware of their fracture. The so small fractures (0.5-1.5 mm) of rotary instruments cannot be observed during preparation and sealing, and without knowing it, these fragments could be exceeded or embedded in the isthmus or on the walls of canals (21).

To the question on how often rotary nickel-titanium instruments should be used before discarding, there can be no definitive answer. Several factors must be considered as the curvature and complexity of the root canal system, the size of the instrument, and the process or method of instrumentation. Given the variability of clinical cases and unpredictability and consequences of fracture, the research is continuing with the help of clinicians and manufacturers to get to know what is the optimal clinical use of endodontic instruments.

To reduce the fracture risk of the instruments within the canal, all files must be examined after each use. All files that show some defects should be discarded immediately. Manufacturing defects can cause fracture of a new instrument even during its first use. Therefore, all instruments must also be examined before each use. Because of minor defects, such as manufacturing errors and plastic deformation, cannot be detected by the naked eye, it is recommended to carry out the examination of the instruments with a magnification of at least 10x.

## Conclusions

Due to the relatively high incidence of deformation of smaller files, these instruments should be considered a single use. To detect small defects or fractures, all instruments should be checked carefully after being employed. It is important not to exceed the maximum number of usages recommended by the manufacturer to reduce the risk of cyclic fatigue, the leading cause of fracture of files (79.07%).
